# Controlled creation and annihilation of isolated robust emergent magnetic monopole like charged vertices in square artificial spin ice

**DOI:** 10.1038/s41598-021-92877-7

**Published:** 2021-06-30

**Authors:** Neeti Keswani, Ricardo J. C. Lopes, Yoshikata Nakajima, Ranveer Singh, Neha Chauhan, Tapobrata Som, D. Sakthi Kumar, Afranio R. Pereira, Pintu Das

**Affiliations:** 1 Department of Physics, Indian Institute of Technology, Delhi, New Delhi, 110016 India; 2 Departamento de Física, Universidade Federal de Viçosa, Viçosa, Brazil; 3 Bio-Nano Electronics Research Centre, Toyo University, Saitama, 3508585 Japan; 4 Institute of Physics, Sachivalaya Marg, Bhubaneswar, Odisha 751005 India; 5 Homi Bhabha National Institute, Training School Complex, Anushakti Nagar, Mumbai, 400 094 India; 6 Present Address: Department of Materials Science and Engineering, Ajou University, Suwon, 16499 South Korea

**Keywords:** Nanoscale materials, Magnetic properties and materials, Magnetic properties and materials

## Abstract

Magnetic analogue of an isolated free electric charge, i.e., a magnet with a single north or south pole, is a long sought-after particle which remains elusive so far. In magnetically frustrated pyrochlore solids, a classical analogue of monopole was observed as a result of excitation of spin ice vertices. Direct visualization of such excitations were proposed and later confirmed in analogous artificial spin ice (ASI) systems of square as well as Kagome geometries. However, such magnetically charged vertices are randomly created as they are thermally driven and are always associated with corresponding equal and opposite emergent charges, often termed as monopole–antimonopole pairs, connected by observable strings. Here, we demonstrate a controlled stabilisation of a robust isolated emergent monopole-like magnetically charged vertices in individual square ASI systems by application of an external magnetic field. The excitation conserves the magnetic charge without the involvement of a corresponding excitation of opposite charge. Well supported by Monte Carlo simulations our experimental results enable, in absence of a true elemental magnetic monopole, creation of electron vortices and studying electrodynamics in presence of a monopole-like field in a solid state environment.

## Introduction

Following the seminal theoretical work of Dirac^1,2^ predicting the existence of a magnetic monopole, search of monopoles has been a major theme in physics^3–6^. The theory shows that the existence of monopole is a precondition for quantization of electronic charge (*e*) which is given by $C_m e=\frac{1}{2}hc$, where $C_m$, *h* and *c* are the strength of a magnetic pole, Planck’s constant and vel. of light, respectively^1,2^. Therefore, the quest for finding signature of stable magnetic monopoles led researchers to investigate in vastly different platforms such as from cosmic radiation to accelerator based high-energy experiments^4,7^. On a different platform, Ray et al. controllably created a nonmagnetic monopole-like state in optical traps using ultra cold rubidium atoms in a Bose Einstein Condesate state^8^. A classical analogue of a monopole was predicted^9^ and experimentally observed^10,11^ in tetrahedral pyrochlore solids where the Ising-like large *f*-electron spins of rare-earth ions interact following a local principle of 2-spins in/2-spins out of tetrahedra, called spin ice rule^12^. There, the monopole is created as a collective behavior of an excitation from a divergence-free low-energy spin ice state which can be considered as vacuum for the local excitation^9^. The emergent monopole in spin ice system carries a magnetic charge and is a non-local entity which is connected to a corresponsing antimonopole by a “string” of spins aligned along one direction thereby maintaining the charge neutrality in the system^10^. These emergent monopole-antimonopole paired states were observed in 2-dimensional analogue of spin ice^13–16^ where dipolar interactions among nanomagnets of strong shape anisotropy mimicking the Ising spin like behavior leads to spin frustration, which in these cases, is by design^17–20^. The excitation of such magnetic charges, which appear to interact via Coulomb-type interaction, are typically observed in large arrays of ASI vertices. They are thermally induced and therefore, random in space and time^16,21,22^. To be useful for fundamental studies or practical applications involving a monopole field, two major challenges are encountered in this case: first, controlled creation and annhiliation of such emergent monopole. Second, stabilisation of an emergent monopole without a corresponding antimonopole. These are also of profound theoretical interest in general^23^. In this work, we investigated the possibility of controlled stabilisation of emergent monopole in isolated square ASI vertices of coordination no. $z=4$ by using external magnetic field as a control parameter. Investigation of finite-size ASI systems, particularly the information on how magnetic charges are formed in finite size systems are very limited in literature^18–20,24^. The work was inspired by the micromagnetic simulations for finite-size-ASI systems involving elliptical-shaped nanomagnets as reported in ref.^24^.

**Figure 1:**
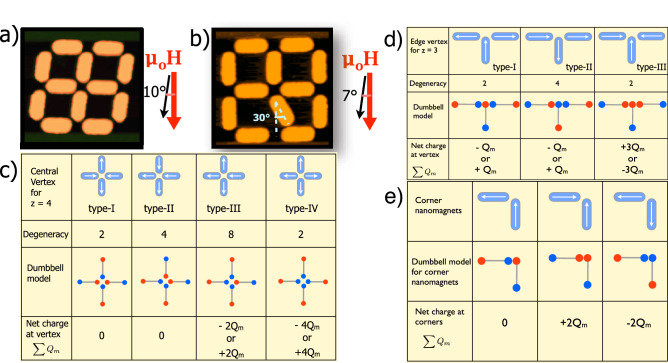
Topography images of ASI systems as well as schematics defining magnetic charges at border and central vertices. AFM images of square ASI vertices with closed edges resembling a stained glass window **(a)** and a deformed stained glass window **(b)**. The stadium shaped nanomagnets are of dimensions $300 \times 100 \times 25$ nm$^3$ and are magnetically in single domain state. In the deformed stained glass window, the misaligned nanomagnet is placed at 30$^{\circ }$ with respect to the long axis of the vertical nanomagnets. The applied magnetic field direction is at an angle of $10^{\circ }$ and $7^{\circ }$ with respect to the vertical nanomagnets for the window patterns **(a,b)**, respectively. **(c)** shows the possible vertex configurations for $z=4$ vertex with nomenclatures of different types (type-I, type-II,...etc.) of spin ice vertices. Schematics of possible orientations of magnetization, magnetic charges in the nanomagnets under dumbbell model and net charges at edge vertices for $z=3$ **(d)** and corners **(e)**.

**Figure 2:**
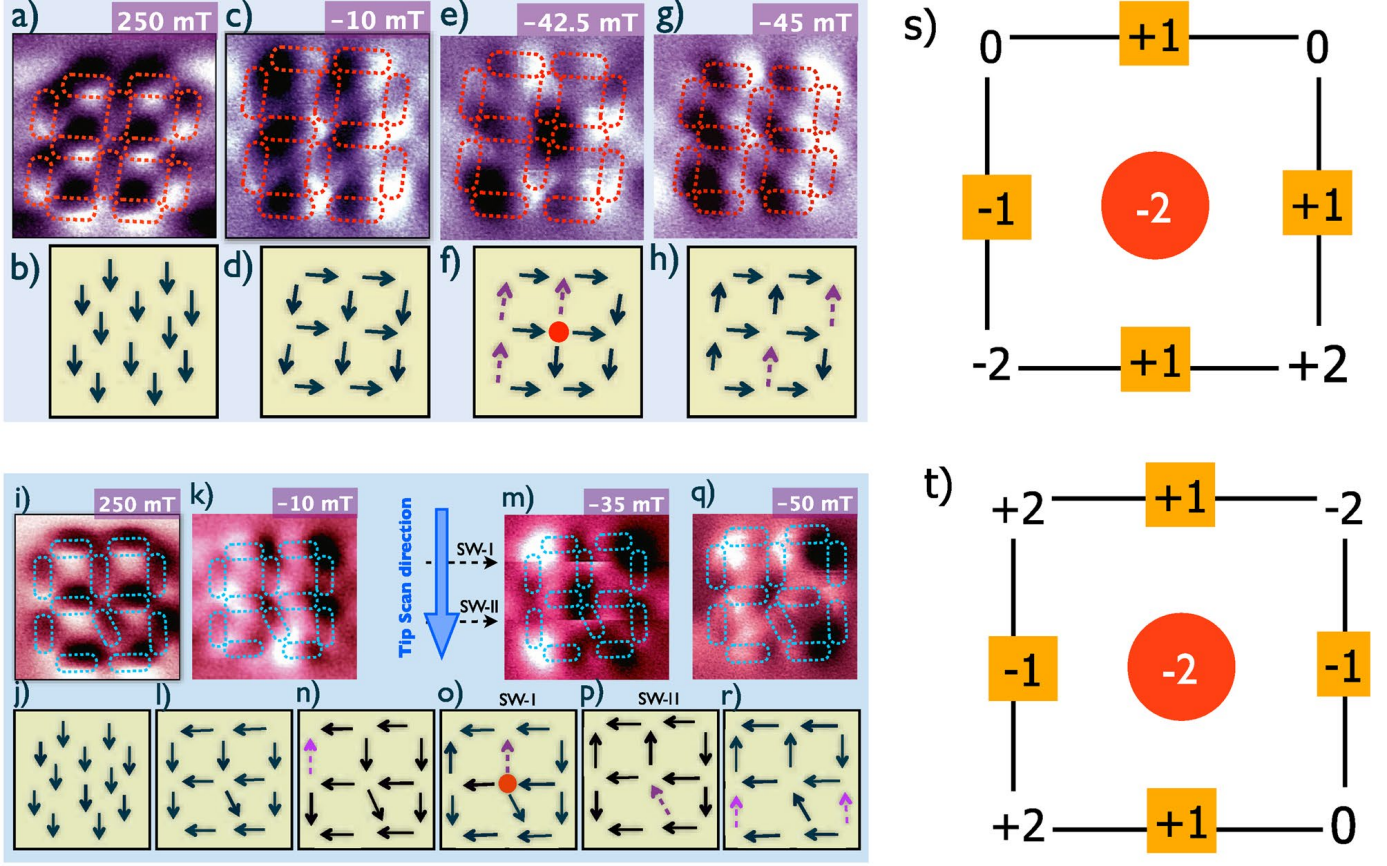
MFM images at discrete magnetic fields and the corresponding arrow diagrams for the two samples (Fig. 1a,b) and description of magnetic charges. **(a,c,e,g)** are MFM images of the stained glass window sample (Fig. 1a) at $\mu _0H_\text{{ext}}=$ 250 mT, − 10 mT, − 42.5 mT and − 45 mT, respectively. **(b,d,f,h)** are corresponding arrow diagrams clarifying the orientation of the net magnetizations in the nanomagnets. The circles at the central vertex position in **(f,o)** indicate emergent monopole-like magnetic charge excitations. **(i,k,m,q)** are MFM images of the deformed stained glass window sample (Fig. 1b) at $\mu _0H_\text{{ext}}=$250 mT, − 10 mT, − 35 mT and − 50 mT, respectively. **(j,l,n–p,r)**, clarifies the orientations of magnetizations at the corresponding fields. The dotted stadium shapes in the MFM images indentify the actual shapes of the nanomagnets and the dotted (magenta) arrows in the arrow diagrams for both samples show the switched nanomagnets at the corresponding fields. **(n–p)** show the three switchings at the bias field of − 35 mT. **(o,p)** are tip-induced switchings (Fig. 2m) at tip scan line positions indicated by SW-I and SW-II while tip scanning in the downward direction. The charge distribution in units of $Q_m$ in the monopole states for undeformed **(s)** and deformed stained glass window **(t)**.

We fabricated isolated vertices comprising of strongly shape anisotropic stadium-shaped nanomagnets of Ni$_{80}$Fe$_{20}$ of dimensions 300 $\times$ 100 $\times$ 25 nm$^3$, in square ASI geometry using electron beam lithpgraphy followed by lift-off process (see “Methods” section). As shown in Fig. 1a, atomic force microscopy (AFM) image of the sample appears as a stained glass window. The nanomagnets are magnetically in single domain state (see MFM images in Fig. 2) and can be treated as switchable macrospins. We used external magnetic field as a control parameter which was applied along the easy axis of one of the islands in the reduced sub-lattice so that there is a finite probability of preferential switching of one vertex-island. In such a vertex where a magnetic charge can exist only as an excitation, a preferential switching of a vertex-island is necessary for the creation of an emergent monopole-like excitation.

## Results and discussions

The magnetization state of the system was probed using magnetic force microscopy (MFM) technique (see “Methods” section). The applied field direction is along [0 $\bar{1}$], practically which lies at an angle of $\sim \,$10$^\circ$ with respect to the easy axis of the vertical nanoislands as shown in Fig. 1a. We first polarized the sample at a field of $\mu _0H_\text{{ext}}=+\,250$ mT (saturation field determined from SQUID as well as micromagnetic simulations is $\sim 170$ mT, see Supplementary Information) and then investigated the response of the nanomagnetic stained glass window by collecting magnetic images at discrete field values while reversing the field. The images were collected at every 2.5 mT so that the complete information of magnetization reversals of the 12 nanomagnets is obtained. Magnetic images collected at saturation (250 mT), − 10 mT, − 42.5 mT and − 45 mT, are shown in Fig. 2a,c,e,g, respectively.

As shown in Fig. 2a, dark and bright patches at the long edges (short edges) of the horizontal (vertical) nanomagnets show that their magnetizations align along the external field direction confirming the saturation at $\mu _0H_\text{{ext}}=250$ mT. This is clarified by a corresponding arrow-diagram, where the arrows show the magnetization direction in the nanomagents (Fig. 2b). While reversing the field, we observe at remanence the magnetization of horizontal nanomagnets, for which the external field is along their hard axes, rotate towards their easy axes whereas that of all the vertical nanonanomagnets are still oriented along the saturation field direction thus producing a 2-in/2-out state at the central vertex which is a spin ice state of type-II (see Fig. 1c). An image collected for $\mu _0H_\text{{ext}}=-\,10$ mT (Fig. 2c) shows the same magnetic state as that of remanence suggesting that no switching has taken place till this field. Considering each dipole of moment $\mu$ as a dumbbell of magnetic charges $\pm Q_m$ (=$\mu /d$) separated by a distance *d*, the central vertex with $z=4$ is at a chargeless ($\sum Q=0$) state^18^. Our MFM data for $-10\,\text {mT} \leqslant \mu _0H_\text{{ext}}\leqslant -\,40$ mT do not show any significant change in the magnetic states of the nanomagnets indicating that no magnetization switching occurs in this field range. At $\mu _0H_\text{{ext}}=-\,42.5$ mT, we observe reversals of bright and dark patches for three vertical nanomagnets suggesting the switchings of their magnetizations (see Fig. 2e,f). These observations suggest that the switchings have taken place within the small field range of − 40 mT$< \mu _0H_\text{{ext}}\leqslant$− 42.5 mT. Our careful analysis indicates that the three nanomagnets switch simultaneously (see below). Interestingly, these switchings lead to the creation of an excited 3-out/1-in state thereby violating the spin ice rule at the central vertex. This excited state at the vertex, which now has a non-zero magnetic charge $\sum Q=-\,2Q_m$, is stable against mutliple scanning by the magnetic tip at the given external field. Thus, a stable isolated emergent monopole-like excitation is created at the central vertex of the stained glass window. In general, in extended arrays of ASI-vertices of similar coordination, magnetically charged vertices are found to occur in pairs of equal and opposite charges (often referred in literature as pairs of emergent monopole and antimonopole) separated by a string of chargeless vertices^15,16,25,26^. This, however, is not the case for vertices with mixed coordination numbers such as for Shakti, Cairo etc. lattices^27–31^. For vertices of mixed coordination, the vertex-charges are screened thereby forming monopole-polaron type bound state^29,31,32^. Typically, the energy cost of separating the oppositely charged monopole–antimonopole like excitations in an array is proportional to the number of chargeless vertices involved between the pair^33,34^. Our experiments reveal that such excited charged magnetic states can be reproducibly stabilised in the absence of any corresponding similarly coordinated vertex excitation of equal and opposite charge. This emergent charge excitation can be annihilated again by external field. We find that upon increasing the field by 2.5 mT, i.e., at a field of − 45 mT, the magnetization of two more nanomagnets switch simultaneously (Fig. 2g,h) leading to the vertex converting to chargeless type-II spin-ice state. Thus, these results demonstrate the controllability of the creation and annihilation of an emergent monopole-like state in the form of a magnetically charged vertex which is remarkable given the reported experimental results so far demonstrated the random creation as a paired monopole–antimonopole state^19,34^.

**Figure 3:**
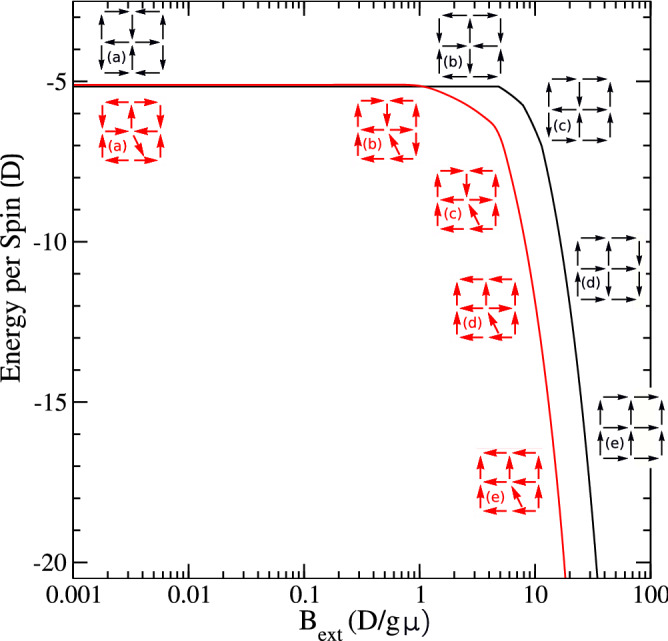
Most probable, minimum energy magnetic configurations for the undeformed (black) and deformed (red) windows plotted against external field. Magnetic field is applied at an angle of 10$^\circ$ with respect to the easy axis of the vertical nanoislands for the former and 7$^\circ$ for the latter sample. Configurations *a* and *b* are the possible ground states for both samples. For undeformed samples, as the external field increases to near $5 D/g \mu$, configuration *b* still shows the most probable state. With further increase of field, configurations *c*, *d* (with a central monopole-like charge excitation) and *e* (type-II) are observed. The same behavior is observed for the deformed window sample.

The controlled creation and annihilation of such isolated emergent monopole-like magnetically charged vertex offers a possibility to utilize them in designing novel experiments. However, it is essential to determine their robustness in such systems. In order to investigate the robustness of such isolated emergent charge excitations against any lattice defect in the vertex, we created an artificial defect with an aim to introduce a perturbation in the dipolar interactions among the vertex-nanomagnets. The defect is in the form of canting of one of the nanomagnets at an angle of $30^{\circ }$ with respect to the vertical nanoislands. Figure 1b shows the AFM image of the defective sample. The canting or misalignment results in a distortion of net local field at the vertex. Except for the misalignment, all other dimensions of the nanomagnets are same as for Fig.1a. The patterned defective structure in Fig. 1b, which can be considered as a deformed stained glass window, was placed in an in-plane external magnetic field such that the field was at an angle of $\sim 7^{\circ }$ with respect to the vertical nanoislands. Calculations based on micromagnetic simulations show that the net dipolar energy of the deformed window is reduced by $\sim$ 18% in comparison with the defect-free vertex as discussed above. This has an intersting consequence in the magnetization switchings as discussed below. The MFM images (Fig. 2i,k,m,q) of the deformed window were collected under the same condition and same field protocol as for the defect-free case discussed above. Similar to the case of undeformed window, here also saturation (at $\mu _0H_\text{{ext}}=\,$250 mT, Fig. 2i) and type-II vertex at remanence are clearly observed. Figure 2k shows the near-remanence data collected at $\mu _0H_\text{{ext}}=\,$ − 10 mT where no switching of magnetization took place so far. The first switching is observed in the MFM image collected at $\mu _0H_\text{{ext}}=\,$− 35 mT (Fig. 2m,n). Additionally, two tip induced switchings are observed at the same external field which are due to the magnetostatic interactions between stray fields emanating from the magnetic tip and the respective nanomagnets. The external field induces the first switching which occurs for an edge-nanomagnet exemplified in the arrow diagram in Fig. 2n. We find that the 1st switching field in this case is reduced by about 18% compared to the defect-free case as discussed above. Interestingly, this is of the same order by which the net dipolar interaction energy is reduced suggesting that such defects may be used as a tuning parameter for controlling switchings in such strongly dipolar coupled systems. We note here that the magnetic configuration at the vertex after this switching remains in 2-in/2-out spin ice state (Fig. 2n). Further interesing observations are made as the magnetic tip scans over the deformed window. As scanning proceeds (downward in the image in Fig. 2m), the magnetization of one of the vertex-nanomagnets suddenly switches (see arrow diagram Fig. 2o). The corresponding nanomagnet which showed dark-bright patches at the edges is now observed as dark–dark patches suggetsing that the nanomagnet’s magnetization switched almost midway (after dark edge is imaged) during the scan. This tip-induced switching due to the local tip-field ($\sim 20$ mT) creates an excited 3-out/1-in magnetically charged emergent monopole-like state at the vertex. As the tip continues to scan further downward and reaches the canted nanomagnet, it prompts the 3rd switching at the same external field (Fig. 2p) which brings the vertex back to the chargeless spin ice state. These tip-induced switchings at the same external field of − 35 mT, clearly indicate the lowering of the barrier energy for switching due to the introduction of the canted defect. Multiple scans over the sample led to the same result demonstrating excellent reprodicibility of the results. These observations demonstrate that the intrinsic magnetization dynamics of the individual nanomagnets and their mutual interactions at an external field of − 35 mT results in to a metastable magnetically charged monopole-like state which can be created and annhiliated by the stray field from the cantilever tip. We believe that this charge excitation at vertex can be stabilised in an external field which has been offset by the tip’s magnetic field. Indeed, in a separate experiment with a cantilever tip of low magnetic moment, a stable monopole-like state was created at a larger field (not shown).

Thus, we find strong experimental evidence for robust isolated emergent charge excitation in finite-size $z=4$ square ASI vertices which can be stabilised without any corresponding oppositely charged vertex. In order to understand how the charge neutrality is reconciled in the isolated charged state of the vertices, we analyze the magnetic charges at the central vertex as well as the edges. The four vertices with $z=3$ at the edges are in either 2-in/1-out or 2-out/1-in state and are naturally charged with absolute value of $\sum Q_\text{{edge-vertex}}=Q_m$ (see Fig. 1d) whereas the absolute value of net charges at the corners are either $\sum Q_\text{{corner}}=2Q_m$ or $\sum Q=0$ (Fig. 1e). 3-in/0-out or 3-out/0-in state causing charge of $\pm 3Q_m$ at $z=3$ vertex is energetically unfavorable. Considering the central vertex as well as all the border charges, we find that both the windows exhibit zero net magnetic charge ($Q_\text{{TOTAL}}$) after each switching. Thus, although the excited vertex is of charge $\sum Q= 2Q_m$, the charges at the boundary arrange themselves so that $Q_\text{{TOTAL}}= Q_\text{{central-vertex}}+Q_\text{{boundary}}=0$ suggesting that the net magnetic charge of both the stained glass window type samples remain conserved at the emergent monopole-like state even in the absence of a corresponding antimonopole-like state. The total magnetic charge distribution at the charge excited state for undeformed window (Fig. 2f) and deformed window (Fig. 2o) are shown in Fig. 2s,t, respectively.

We next investigated the validity of this remarkable experimental realization of isolated emergent monopole-like state in stained glass window samples theoretically by performing Monte Carlo simulations at different external fields ($\mathbf {B_\text{{ext}}}$). By considering the exact field configuration as in the experiments for both samples, we calculated the corresponding energy and probability for all possible states from which we determined the most probable magnetic configuration for the two systems at room temperature (see Videos 1, 2 in Supplementary Material). As shown in Fig. 3 (black curve) where energy per spin is plotted against $B_\text{{ext}}$, the configuration of the interacting macrospins in the undeformed stained glass evolves as follows: firstly, for very low field ($B_\text{{ext}} \approx 0$), the degenerate ground state (type-I vertices of configurations *a* and *b* in the figure with antiferomagnetic ordering of macrospins) is observed. However, for $B_\text{{ext}} \sim 1D/g \mu$, the degeneracy is broken as the configuration *b* becomes energetically more favorable. Here, *D* is the dipolar interaction constant, *g* is the gyromagnetic factor and $\mu$ is the net average magnetic moment of the nanoislands. As the field increases further from $1D/g\mu$, the configuration *c* is stabilised. Upon further increase of field, a magnetically charged vertex (configuration *d*) becomes energetically most stable for $B_\text{{ext}}\sim 20D/g\mu$. The charged vertex remains stable till about $B_\text{{ext}}\sim 25D/g\mu$ before it converts to a chargeless type-II state (configuration *e*). The system then saturates at a high field (not shown). Similar behavior is observed for the deformed stained glass-like sample (red curve in Fig. 3) where the field is applied at an angle of 7$^{\circ }$ in order to match the experimental conditions. Interestingly we find that the corresponding configurations for this case occur at reduced energies which is also observed in the experiments as discussed above. The emergent monopole-like state for this sample is observed at a relatively lower field $B_\text{{ext}}\sim 10D/g\mu$ which again changes to type-II chargeless vertex state at $B_\text{{ext}} \sim 15D/g\mu$ (Fig. 3). Quantitatively, considering the average magnetic moment of each nanoisland $\mu \sim 9.65\times 10^{-12}$ emu (as experimentally determined from the saturation magnetization, see Supplementary Material), we find dipolar interaction energy, $D\approx 3.86\times 10^{-15}$J for the undeformed samples whereas $D\approx 6.76\times 10^{-15}$J for the deformed sample. For the calculations, the monopole fields of 40 mT and 35 mT, respectively were considered for the undeformed and deformed samples, respectively. Thus, our experimental findings of emergent monopole-like charged vertex state in conjunction with Monte Carlo simulations provide an estimation of the dipolar interaction energy.

The analysis of magnetic charges observed in our Monte–Carlo simulations demonstrate a clear resemblance with the experimental results underlining the observation that the net magnetic charge is conserved at the emergent monopole-like state (see Supplementary Material). The monopole-like excitations at the centre of the vertices are screened by the magnetic charges at the borders. Thus, we find a clear evidence that the conservation of magnetic charges is a precondition for stabilisation of isolated monopole-like excitation in square ASI vertices. We note here that any change in magnetic charge at the boundary (surface) annihilates the corresponding charge state at the vertex (in bulk). Thus, our experimental observations of the stabilisation of isolated emergent monopole-like states are in excellent agreement with the Monte–Carlo based calculations which delineate a clear condition for creating such excited states in finite-size ASI systems. The results also suggest that the creation of emergent monopole–antimonopole pair is not a necessary requirement for preserving the charge neutrality in a large array of ASI vertices. The neutrality may be locally conserved through the border charges as we observe in our studies. This precondition implies that the multipole expansion for the potential of the stained glass *ASI* containing the central monopole-like excitation has a null monopole term and the whole system of the vertex and the boundaries must possibly contain an expansion in terms of dipole, quadrupole, etc. i.e., $V(r) \sim 1/r^{2} + 1/r^{3}+\cdots$. Therefore, we infer that it is impossible to create an excited charged vertex for an isolated open-edged square-ASI vertex with $z=4$. Since there is no edge spins to compensate the charge at the vertex, only chargeless spin ice state of type-I or type-II can be stabilised with external magnetic field.

**Figure 4:**
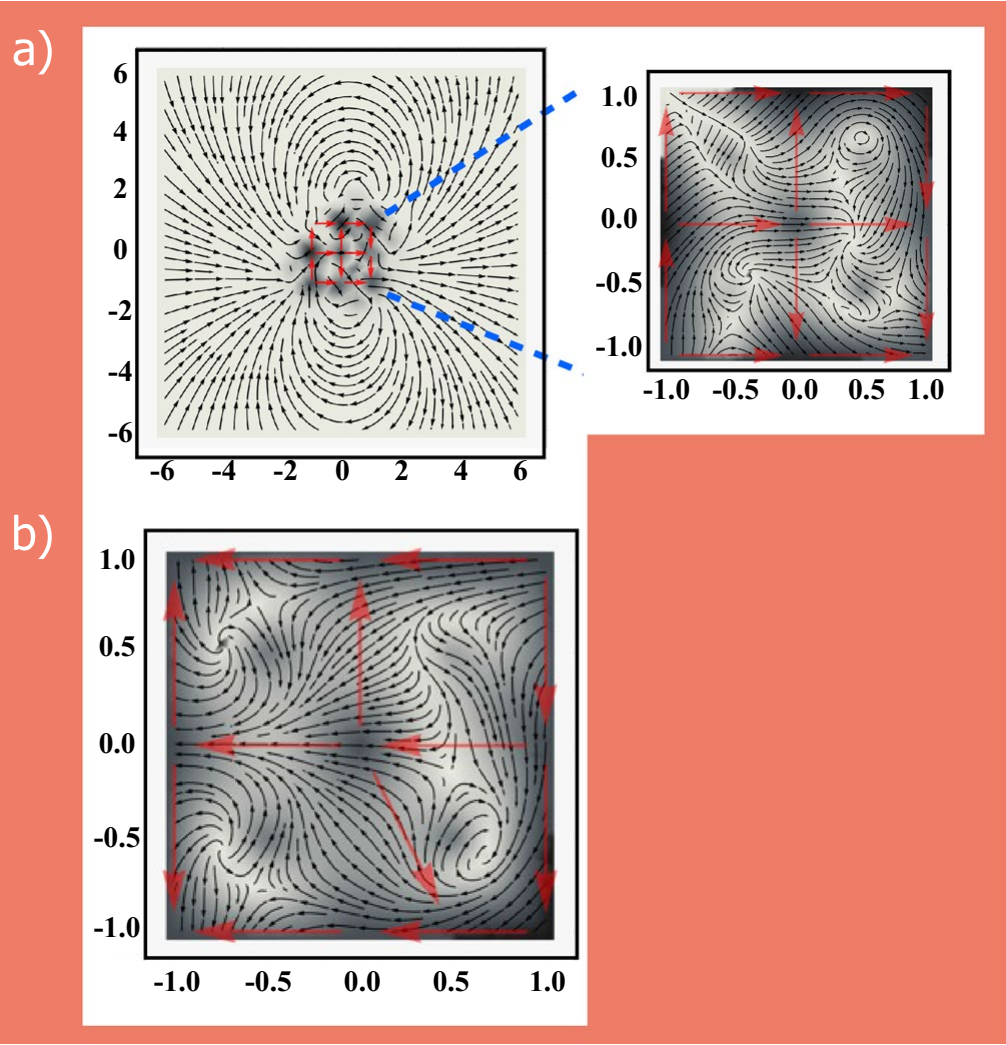
Magnetic field lines for stained glass (**a**) and deformed stain glass window (**b**) samples. (**a**) Shows the far-field image data. The inset exemplifies the zoomed near-field data. (**b**) Shows near field data of the deformed window. The length scales for both figures are in units of the lattice constant of the artificial lattice.

To gain insight into the exact behavior of magnetic field lines, particularly at the monopole-like charged vertex states, we performed calculations (see “Methods” section) to determine the magnetic field lines for such charged vertices in our stained glass samples. Figure 4 shows the field lines for the two samples. Detailed analysis of these results show that the system as a whole resembles the behavior of a magnetic dipole ($\mathbf {\nabla }\cdot \mathbf {B}=0$), however, the emergent monopole like behavior is observed as a local effect at the central vertex where $\mathbf {\nabla }\cdot \mathbf {B}\ne 0$ (Fig. 4a). This is consistent with our analysis of the magnetic charges where we observe the net zero charge for the entire system which is conserved at the emergent monopole state. Similar behavior of local field lines is observed for the deformed window at the monopole-like state (Fig. 4b). Except for the differences in the local field lines near the edges, the overall behavior exhibited by the deformed and undeformed samples are expectedly similar. These observations of charge neutrality at the monopole-like states are further confirmed by the Monte Carlo analysis of a broken stained glass window where the 50% of the edge islands are removed from the undeformed structure (see Supplementary Mateial).

## Conclusion

The isolated emergent monopole-like charged vertex states that we observe in these finite-size ASI systems is a classical entity and does not obviously solve Dirac’s quantum mechanical problem. However, our work demonstrates how charges in a *finite-size* square ASI system are formed at the central vertex with redistribution of charges at the edges thereby maintaining the charge neutrality at the single $z=4$ vertex level unlike for most extended arrays of ASI vertices where paired charged vertices are observed. The central charge at $z=4$ vertex is always screened by the distributed charges at the borders.

The results have significant theoretical ramifications. The demonstration of the *controlled* stabilisation of isolated emergent monopole-like charge state is a significant result particularly since Dirac or Nambu type energetic strings in arrays of spin ice vertices are currently under debate. It would be interesting to study the controllability of such charged vertices in an extended geometry (array). Due to the local variations in the dipolar coupling between the nanomagnets or due to variations in magnetization switching barrier parameters, a significant distribution of the switching fields in large arrays is expected. It may be possible to controllably generate a single or a few charged vertices in large arrays. Also, it remains to be explored if in degenerate spin ice systems, achieved by equalizing the coupling strengths $J_1$ between perpendicular and $J_2$ between collinear islands using height-offset to one sublattice^26,35^, such controlled magnetic charge excitations can be generated. If it is possible to generate controlled charge excitations in an extended array, then these charge states may act as potential sources of pinning for the Faraday lines identifying domains in the extended array. In that case, it may be possible to study interesting kinetics of the Faraday lines (or domain walls) as recently suggested by Nisoli et al.^36^. Our analysis also suggests revisit of the concept of the charge neutrality in extended large arrays of the ASI systems with same coordination where monopole-antimonople-like pairs are created randomly.

In practical terms, our results could pave the way for a few applications. Plane wave electrons traversing through isolated monopole field acquire azimuthal component due to Aharonov–Bohm effect thereby creating vortex beams of electrons^37^. Interaction of such vortex beams of electrons with matter has potential to lead to multiple applications. Our work suggests that such isolated emergent magnetic charge excitations with diverging fields, if controllable at remanence, may be useful to explore creation of electron vortex beams in a controlled way. Also, controlled diverging field at such ASI vertices may enable investigating the effect of scattering of a Dirac particle with charge *Ze* at relativistic^23^ or nonrelativistic^38^ velocities by an infinitely heavy magnetic charge excitation (fixed monopole-like field). We speculate that such experiments based on the isolated emergent magnetic charges may be possible to test the prediction of helicity flip of Dirac particles scattered off fixed magnetic monopole-like field^23^.

## Methods

### Sample fabrication

Our nanomagnetic islands of Ni$_{80}$Fe$_{20}$ were fabricated on Si with a native oxide layer using bilayer resists stack comprising of ZEP520A (positive type) and PMGI-SF3. The patterns were defined using Elionix ELS-G125TY e-beam lithography writer. The exposed ZEP520A resist was developed in o-Xylene. The PMGI resist which was not covered by ZEP520A was etched using NMD-3 solution. The sample was then O$_2$ plasma-cleaned before starting e-beam deposition in an evacuated chamber with a base pressure of $5\times 10^{-7}$ mbar. A Ti layer of thickness 7 nm was deposited on SiO$_2$ to increase the adhesion of the magnetic permalloy (Ni$_{80}$Fe$_{20}$) layer on the surface. An Al llayer of thickness 5 nm was used as a capping layer to prevent oxidation of Ni$_{80}$Fe$_{20}$. The entire electron beam deposition was carried out without breaking the vacuum. The Ti and Al layers were deposited at a rate of 1Å/s whereas Ni$_{80}$Fe$_{20}$ layers were depositied at 0.8Å/s at a base pressure of $5\times 10^{-7}$ mbar, respectively. The liftoff process was carried out using tetrahydrofuran (THF) bath followed by solvent Remover PG (make: MicroChem). For further details, see Supplementary Material.

### MFM measurements

Magnetic imaging of the stained-glass window samples were carried out using a commercial magnetic force microscope (make: Asylum Research, model: MFP-3D). The MFM system is fitted with a permanent magnet fixed on a rotation module which allowed the in-plane field to vary in the range of $\pm 250$ mT. Data were collected using Co-Cr coated tip (ASYMFM, Asylum Research) commercially procured from Asylum research. The tip’s average magnetic moment is $1\times 10^{-13}$ emu.

### Monte–Carlo simulations

For Monte–Carlo simulations, the same field configuration as used in the experiments, i.e., the external field for stained window glass sample was applied at an angle of 10$^\circ$ with respect to the easy axis of the vertical nanoislands (except that here the field is applied upward, which does not affect for a square lattice). For the deformed stained glass sample, likewise, the external field was applied at an angle of 7$^\circ$ with respect to the easy axis of the vertical nanoislands. The exact misalignement angle of 30$^\circ$ was considered for the respective nanomagnet. All the simulations were performed for room temperature and varying magnetic field. We calculated the energy and probability for all $2^{12}$ possible configurations by using a canonical ensemble at varying $\mathbf {B}$ (see supplementry material). From these calculations we constructed the energy histograms for a range of $\mathbf {B}$ and determined the most probable magnetic configurations obtained at different $\mathbf {B}$ values.

### Calculations of magnetic field lines

The magnetic field lines for the system excitations are obtained by subtracting the magnetic field of the fundamental state ($\mathbf {B}_\text{{fund}}$) from that of the excited state ($\mathbf {B}_\text{{exc}}$) i.e., $\Delta \mathbf {B}=\mathbf {B}_\text{{exc}}-\mathbf {B}_\text{{fund}}$. $\mathbf {B}_\text{{fund}}$ is the field for magnetic configuration (a) in Fig. 3 for both samples. From this vector field, we draw the tangent lines that follow the same vector field direction. The length scales used are in the units of lattice constant of the artificial lattice.

## Supplementary Information

Supplementary Video 1.

Supplementary Information.

Supplementary Video 2.
