# Supplementary material for: Controlled creation and annihilation of isolated robust emergent magnetic monopole like charged vertices in square artificial spin ice
Source: Sci Rep. 2021 Jun 30;11:13593. doi: 10.1038/s41598-021-92877-7 (PMC8245615; doi:10.1038/s41598-021-92877-7)
Supplement: Supplementary file 2 — Supplementary Information. [file 41598_2021_92877_MOESM2_ESM.pdf]

Controlled creation and annihilation of isolated robust emergent magnetic monopole like charged vertices in square artificial spin ice

N. Keswani, *et al.*

May 19, 2021

Supplementary Material

Sample fabrication

For the patterning of our nanomagnetic islands, we used bilayer resists PMGI-SF3 and ZEP520A (positive type). The Si/SiO_2 substrate was spin coated first with PMGI resist at 500 rpm for 5 s and 3000 rpm for 30 s and was prebaked at 200°C for 10 min using a hotplate. The second layer resist ZEP520A was then spin coated at 500 rpm for 5 s and 3000 rpm for 60 s and then prebaked at 180°C for 3 min on a hotplate. The e-beam lithography (EBL) system ELS-G125TY (make: ELIONIX Inc., Japan) was used to define the patterns with a 125 keV electron beam at a dose of 300 μC/cm^2. The exposed ZEP520A was developed in o-Xylene for 3 min and then rinsed in isopropyl alcohol (IPA) for 1 min. Thereafter the samples were treated in O_2 plasma generated by applying 100 W radio frequency power at a pressure of 15 Pa for 10 s to change the surface of the ZEP520A resist from hydrophobic to hydrophillic nature. This helps easy flow of NMD-3 solution which was used to etch (for 20 s) the part of the PMGI resist that was not covered by ZEP520A. The samples were then rinsed in deionized (DI) water for 1 min. The samples were again rinsed with methanol and isopropyl alcohol (IPA). A second O_2 plasma treatment was carried out to ensure a clean surface prior to deposition. After following the proper resist development procedure, the samples were immediately kept inside an evacuation chamber for e-beam deposition of the metallic films. A Ti layer of thickness 7 nm was deposited on the patterned SiO_2 to increase the adhesion of the magnetic permalloy ($Ni_{80}Fe_{20}$) layer on the surface. An Al layer of thickness 5 nm was used as a capping layer to prevent oxidation of the magnetic layer. The Ti, $Ni_{80}Fe_{20}$ and Al layers were deposited at the base pressure of 5×10^{-5} Pa. The deposition rates for Ti and Al were 1 Å/s and that for $Ni_{80}Fe_{20}$ was 0.8Å/s. The entire electron beam deposition was carried out without breaking the vacuum. After the deposition, the lift-off process was used to obtain the patterned structures. For the lift-off, the samples were placed in tetrahydrofuran (THF) bath till the resist dissolved. The samples were then washed in IPA and were immersed in a solvent, Reomver PG (make: MICROCHEM), at 50°C on hotplate until the PMGI dissolved completely. The samples were finally cleaned with DI water.

Measurements for average magnetization of individual spin ice vertices

For the estimation of dipolar interaction constant D for our undeformed and deformed stained window glass samples, values of average magnetic moment of individual $Ni_{80}Fe_{20}$ nanomagnets are required. We experi-

Parameters	Stained glass window	Deformed stained glass window
Total no. of nanomagnets	1555200	1555200
Net magnetic moment at saturation, m_s (emu)	$\sim 1.5 \times 10^{-5}$	$\sim 1.5 \times 10^{-5}$
Saturation field (H_s)	$\sim 195\,\mathrm{mT}$	$\sim 195\,\mathrm{mT}$
Coercive field (H_c)	$\sim$10 mT	$\sim$10 mT

Table 1: Table summarizes the different parameters obtained from global magntization measurement data as shown in Fig. S1. The global measurements show nearly identical behavior for both the samples.

mentally determined the average magnetic moment of individual magnetic nanoislands in both finite size ASI samples. Direct magnetic measurements of such individual nanomagnets require high-sensitive probe such as dc-micro SQUID [1], 2-dimensional electron gas based micro-Hall magnetometry etc. techniques [2, 3, 4]. For our measurements, multiple (more than a million) isolated single vertices for both sample types were patterned using EBL so that the measurements could be performed with the sensitivity of SQUID or vibrating sample magnetometer. For convenience of fabrication, we lithographically patterned four separate grids of 180×180 vertices with closed edges. Since each of these individual closed-edge vertex structures has 12 nanomagnets, total no. of nanomagnets patterned is $180 \times 180 \times 4 \times 12$, i.e. 1555200. Fig. S1(a) shows the SEM image of four grids and Fig. S1(b,c) show a part of one of the four grids for stained window sample. The deformed stained glass window sample was patterned in the same way (Fig. S1(d)) .

The measurements of net magnetic moment for these nanomagnets were carried out at room temperature using a vibrating sample magnetometer (VSM) equipped in a physical properties measurement system (PPMS, make: Quantum Design). The magnetic field was applied in-plane. Fig. S1(e) shows the magnetization data observed at room temperature for the two types of samples. From the magnetization measurements, average magnetic moment of the individual nanomagnet was determined by dividing the net magnetic moment at saturation by the total no. of nanomagnets, i.e., 1555200. The average magnetic moment of the individual nanomagnets was estimated to be $\frac{1.5\times10^{-5}}{1555200} = 9.65 \times 10^{-12}$emu. Table-1 shows the parameters obtained from the global magnetic measurements.

MFM measurements:

Magnetic imaging of the stained-glass window samples were carried out using a commercial magnetic force microscope (make: Asylum Research, model: MFP-3D). The MFM system is fitted with a permanent magnet fixed on a rotation module which allowed the in-plane field to vary in the range of $\pm 250\,\mathrm{mT}$. Data were collected using Co-Cr coated tip (ASYMFM, Asylum Research) commercially procured from Asylum research. The tip's average magnetic moment is 1×10^{-13} emu.

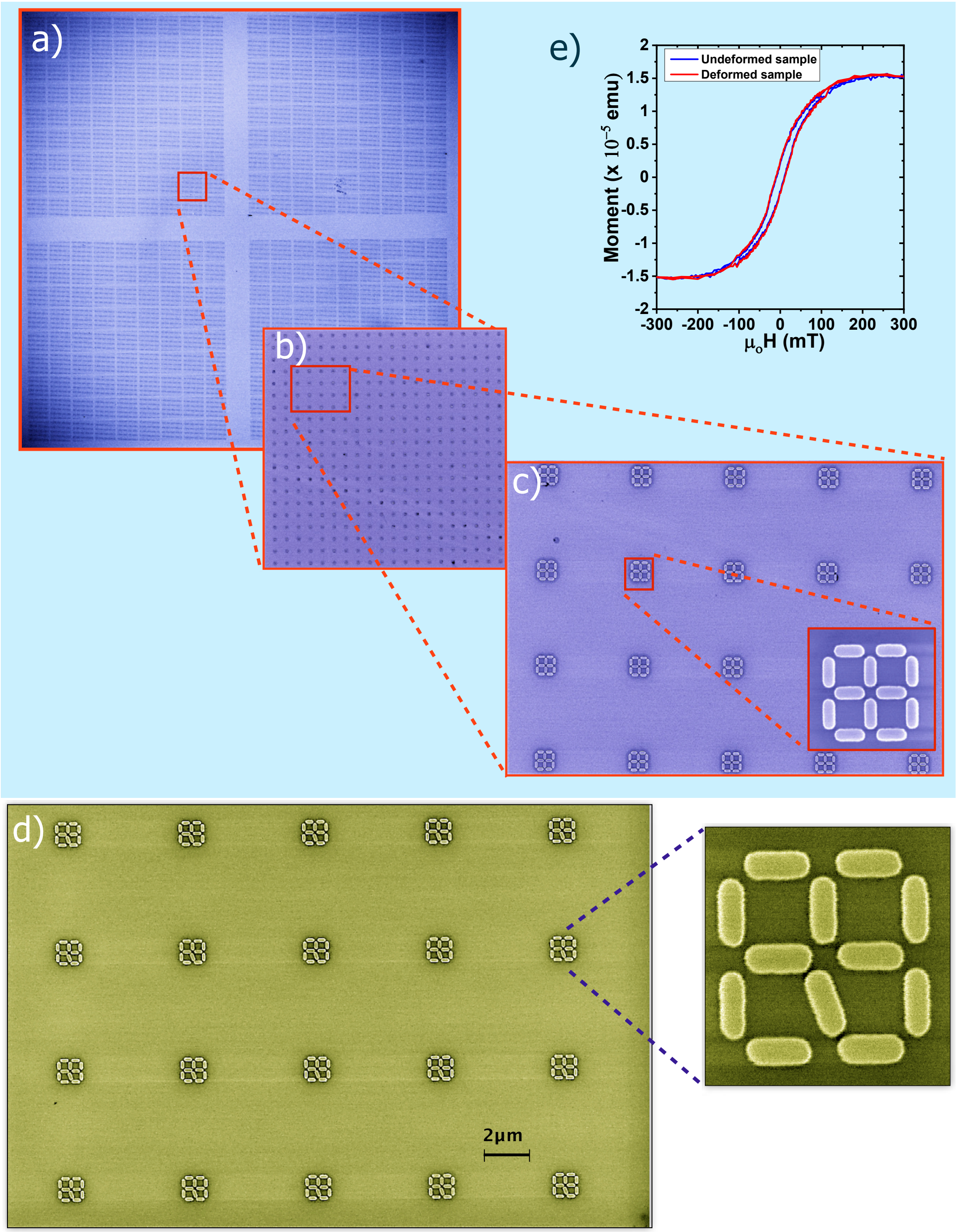

Figure S1: (a)-(c) Scanning Electron Micrograph (SEM) data for undeformed stained glass window samples. Individual patterns in the four grids for undeformed samples are shown by the zoomed-in images (b-c). Deformed stained glass window samples were similarly patterned. (d) SEM micrograph for a part of the deformed stained glass window sample. (e) M-H graphs for both types of samples are shown in (top) right.

Monte Carlo simulations:

For Monte Carlo simulations, the same field configuration as used in the experiments, i.e., the external field for stained window glass sample was applied at an angle of 10° with respect to the easy axis of the vertical nanoislands (except that here the field is applied upward, which does not affect for a square lattice). For the deformed stained glass sample, likewise, the external field was applied at an angle of 7° with respect to the easy axis of the vertical nanoislands. The exact misalignement angle of 30° was considered for the respective nanomagnet. All the simulations were performed for room temperature and varying magnetic field. We calculated the energy and probability for all 2^{12} possible configurations by using a canonical ensemble at varying $\vec{B}$ (see supplementry material). From these calculations we constructed the energy histograms for a range of $\vec{B}$ and determined the most probable magnetic configurations obtained at different $\vec{B}$ values.

Calculations of magnetic field lines:

The magnetic field lines for the system excitations are obtained by subtracting the magnetic field of the fundamental state ($\vec{B}_{\text{fund}}$) from that of the excited state ($\vec{B}_{\text{exc}}$) i.e., $\Delta\vec{B} = \vec{B}_{\text{exc}} - \vec{B}_{\text{fund}}$. $\vec{B}_{\text{fund}}$ is the field for magnetic configuration (a) in Fig. 3 for both samples. From this vector field, we draw the tangent lines that follow the same vector field direction. The length scales used are in the units of lattice constant of the artificial lattice.

Field-evolution of magnetic charges in the Monte Carlo calculations

The magnetic charges for the most probable magnetic configurations at different external magnetic fields as obtained by Monte Carlo calculations (Fig. 3 in main text) are analysed according to the the dumbbell model of a magnetic dipole. According to the charge description shown in Fig. 1(c-e) in the main text, we find that both deformed and undeformed samples remain magnetically neutral for all configurations before the system saturates. Fig. S2 shows that the net zero magnetic charge for each magnetic configurations remains conserved during the creation and annihilation of isolated emergent monopole state.

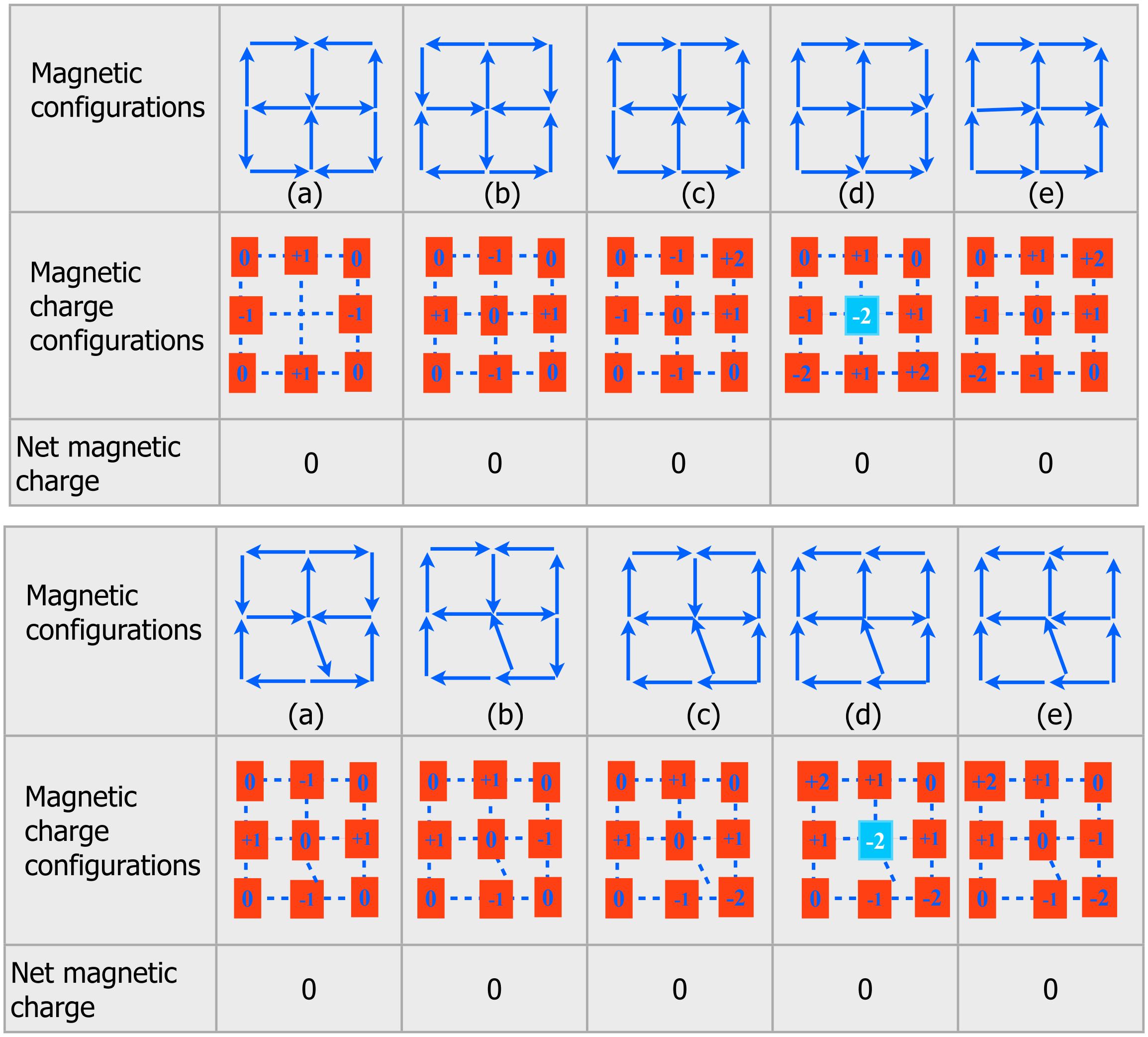

Figure S2: Most probable magnetic configurations (shown by arrows) of nanoislands at room temperature and corresponding magnetic charges for (top) stained glass window sample and (bottom) deformed window sample. Monopole central vertices are shown by blue squares. Other charges are shown by red squares. The magnetic configurations are Monte Carlo simulated results as shown in Fig. 3 in the main text.

Video of energy histograms

The videos show the probability of all configurations and their energies, within a canonical ensemble. Individual videos (file names: i) Undeformed-sample.mov and ii) Deformed-sample.mov) for the corresponding two different samples are constructed as follows: first, for a given applied external magnetic field $\vec{B}_{\text{ext}}$, the energies for all 2^{12}

possible configurations is calculated. All the energies are shown in the turquoise graph (in video), normalized by the maximum energy of the system for that applied field. It is important to note that each configuration is previously labeled from 1 to 2^{12}. Then, the partition function $\mathcal{Z} = \sum_{i=1}^{4096} e^{\beta E_i}$, for this finite case is obtained. Next, the probability for each configuration, $P_i = e^{\beta E_i}/\mathcal{Z}$, is calculated and plotted on the graph (red curve). This process is repeated for each value of the applied field and the graph-video is then updated. It is also important to note that the most likely states within a given canonical ensemble provide us with an estimate of the possible states which are observed in the actual experiment. In our case, there is an agreement between the most likely states and the experiment for temperatures near room temperature. The two degenerate most probable states for initial fields are seen as two configurations of probabilities ~ 0.5. The final configuration with probability 1 is the state when the system saturates at high external fields. We note here that although the videos show the most probable states (different magnetic configurations) which are most likely to be observed in the experiments, a discrepency in the corresponding exact $\vec{B}_{\rm ext}$ values in the videos and calculations of the energy per spin versus $B_{\rm ext}$ (Fig. 3 in main text) arises due to the use of finite partition functions used for calculations for preparing the videos. The finite partition functions were used to calculate the probability of accessing a state within the ensemble. For the calculations of energy vs $B_{\rm ext}$ (Fig. 3 in main text), Boltzmann-type weight was used to calculate the probability which in this case is more realistic. The videos are presented only to show the field-dependent most propbable accessible states (i.e., magnetic configurations) for the two samples. The vidoes were generated using GNUPlot-x11 and FFmpeg.

Monte Carlo simulation for a broken window glass

As a further test of the precondition of the emergent monopole behavior, Monte Carlo calculations were performed for another type of defective vertex where 50% of the edge-islands were removed (thereby forming a kind of a broken stained glass window) so that there is a charge imbalance in the border charges (see Fig. S3). In the broken glass window, we again observe a type-I ground state (state a). The calculations show an excited state for a very small magnetic field for $B_{\rm ext} \sim 2.5D/g\mu$ (state (b) In Fig. S3). Even in the presence of the charge imbalance of border charges for this case of broken glass window, the magnetizations of respective nanoislands are found to orient such that the net zero magnetic charge of the whole system is maintained at the monopole state. Thus, this provides an additional test of the condition for creation of emergent monopoles in individual vertices.

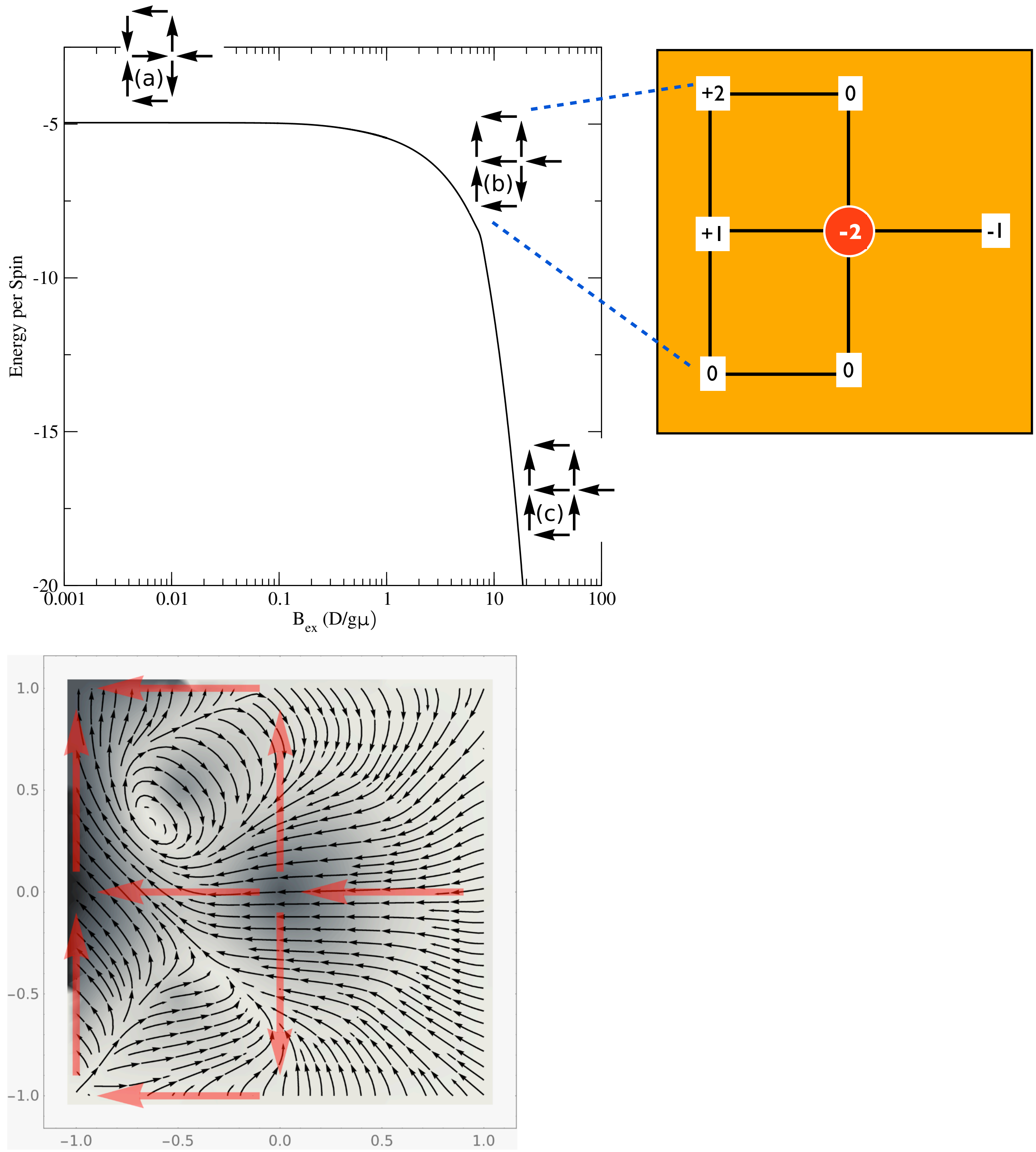

Figure S3: (top) Monte Carlo simulation results for 50% broken glass window. The energy per spin is plotted in units of dipolar interaction term D (see Fig. 3 in main text). The ground state 'a' changes to an excited state 'b' in presence of external field. State 'c' is a type-II spin ice state. The figure in right shows the distribution of the magnetic charges according to the dumbbell model at the monopole state. The net magnetic charge remains zero through out the transitions. (bottom) 2-dimensional magnetic field lines for the emergent monopole state. The length scale for both x and y axes is in the unit of the lattice constant of the artificial lattice.

References

[1] Wernsdorfer, W., Doudin, B., Mailly, D., Hasselbach, K., Benoit, A., Meier, J., Ansermet, J. -Ph. & Barbara, B. Nucleation of Magnetization Reversal in Individual Nanosized Nickel Wires. *Phys. Rev. Lett.* 77, 1873 (1996).

[2] Pohlit, M., Porrati, F., Huth, M., Ohno, Y., Ohno, H., & Müller, J. Magnetic stray field studies of a single cobal nanoelement as a component of the building blocks of artificial spin ice. *J. Mag. Mag. Mat.* **400**, 206 (2016).

[3] Keswani, N., Nakajima, Y., Chauhan, N., Ukai, T., Chakraborti, H., Gupta, K. D., Hanajiri, T., Kumar, S., Ohno, Y., Ohno, H., & Das, P. Complex switching behavior of magnetostatically coupled single domain nanomagnets probed by micro-Hall magnetometry. *Appl. Phys. Lett.* **116**, 102401 (2020).

[4] Li, Y., Xiong, P., Von Molnàr, S., Wirth, S., Ohno, Y. & Ohno, H. Hall magnetometry on a single Fe nanoparticle. **80**, 4644 (2002).
